# Decompensated Heart Failure as the Initial Presentation of Multiple Myeloma: A Case Report

**DOI:** 10.7759/cureus.29658

**Published:** 2022-09-27

**Authors:** Nodari Maisuradze, Nazeera Ghanie, Adam Kurnick, Micha Gooden, Rafsan Ahmed

**Affiliations:** 1 Internal Medicine, State University of New York Downstate Medical Center, Brooklyn, USA

**Keywords:** al amyloidosis, echocardiography, multiple myeloma, cardiac amyloidosis, heart failure

## Abstract

Amyloid deposition in the setting of multiple myeloma (MM) is a well-documented phenomenon. In this paper, we present the rare case of a 62-year-old male who presented with decompensated heart failure in the setting of cardiac amyloid deposition as the initial presentation of MM. The patient presented to the emergency department with two weeks of worsening lower extremity edema. Laboratory exam revealed elevated troponin I, elevated B-type natriuretic peptide (BNP), macrocytosis, increased urine protein/creatinine ratio, and a monoclonal peak on both serum protein electrophoresis (SPEP) and urine protein electrophoresis (UPEP). Transthoracic echocardiogram (TTE) revealed findings suggestive of amyloidosis. Abdominal fat pad biopsy confirmed amyloid deposition. The patient did not have other symptoms typically seen in multiple myeloma, such as fatigue or weakness, bone pain, or weight loss. In conclusion, we present a rare case of decompensated heart failure in the setting of amyloidosis as the initial presentation of multiple myeloma.

## Introduction

Multiple myeloma is a plasma cell malignancy that accounts for a large percentage of all hematologic cancers [1]. It is associated with a clonal plasma cell proliferation in the bone marrow leading to monoclonal protein in the blood and associated end-organ dysfunction [2]. Common presenting symptoms include generalized weakness, anemia, bone pain or pathological fractures, renal impairment [2]. In addition, some patients with multiple myeloma may develop clinically significant immunoglobulin light chain (AL) amyloidosis [3]. AL amyloidosis involves multiple organ systems, commonly affecting the kidneys with the second most common presenting manifestation being cardiac [4]. Pathophysiology of AL cardiac amyloidosis involves the deposition of monoclonal Ig light chains as insoluble proteinaceous fibrils in the extracellular space of the heart [3, 4]. Amyloid deposition leads to expansion of the extracellular space in the heart without a compensatory ventricular dilation resulting in restrictive pathophysiology [4]. We present a rare case of a patient presenting with decompensated heart failure due to AL cardiac amyloidosis with associated multiple myeloma without any other clinical features of multiple myeloma.

## Case presentation

A 62-year-old man with a past medical history of hypertension presented to the emergency department complaining only of five days of progressive bilateral lower extremity edema. Upon questioning, he denied chest pain, shortness of breath, orthopnea, paroxysmal nocturnal dyspnea, dyspnea on exertion, change in exercise tolerance, fevers, leg pain, and decreased urinary output. His vital signs were within normal limits. Physical examination revealed bilateral, symmetric, nonerythematous, nontender lower extremity pitting edema up to the midshins. His lungs were clear to auscultation bilaterally. The remainder of the physical exam was unremarkable.

**Figure 1:**
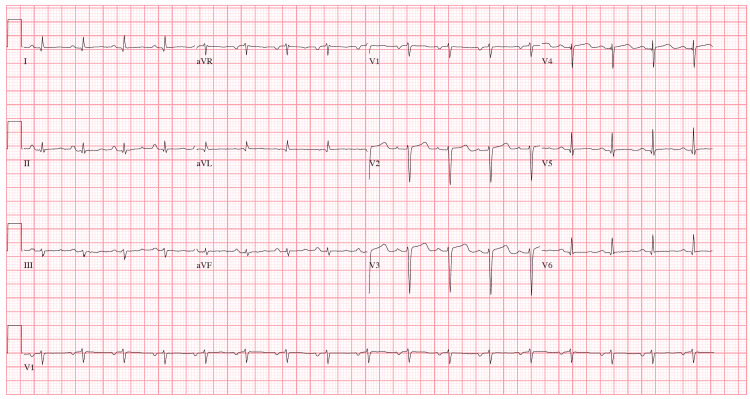
12-lead EKG demonstrating low voltage in limb leads and left atrial enlargement

**Figure 2:**
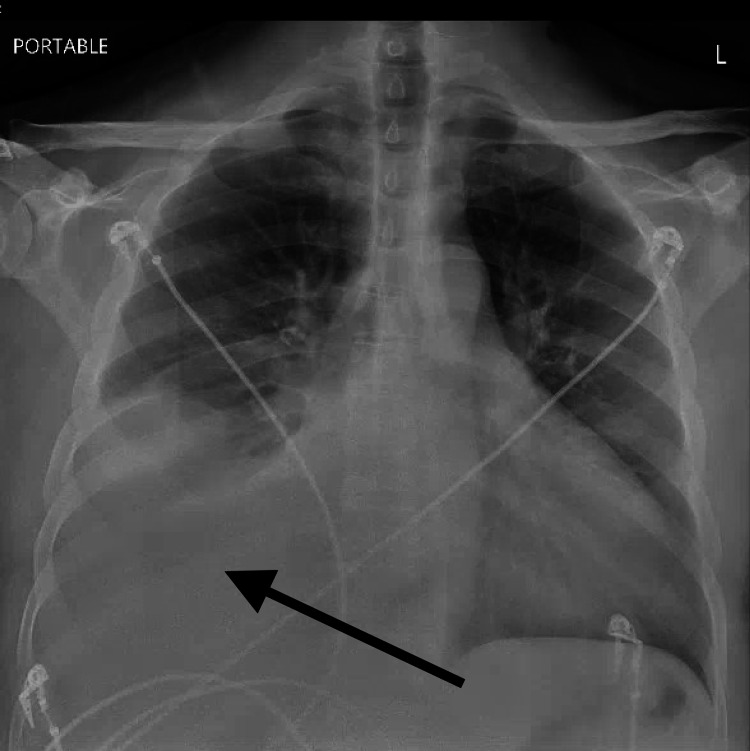
Chest X-Ray demonstrating right-sided pleural effusion Black arrow pointing to a pleural effusion

His EKG demonstrated a normal sinus rhythm, low voltage in limb leads and signs of left atrial enlargement (Figure 1). Labs were notable for a B-type natriuretic peptide (BNP) of 2110 pg/ml, troponin I of 2.11 ng/ml with a creatinine of 1.2 mg/dL, an elevated protein gap of 4.9 with a total protein of 8.3 g/dL, and an albumin of 3.4 g/dL. Other notable labs included a calcium of 9.4 mg/dL, a hemoglobin of 14.0 g/dL, and an mean corpuscular volume (MCV) of 114.4 fL. A chest X-ray (Figure 2) was consistent with vascular congestion. The initial assessment was acute decompensated heart failure secondary to an acute non-ST segment elevation myocardial infarction (NSTEMI). The patient underwent an urgent cardiac catheterization that demonstrated nonobstructive coronary artery disease. A transthoracic echocardiogram (TTE) showed a reduced left ventricular ejection fraction (LVEF) to 40% with apical sparing of global longitudinal strain (GLS) and mild wall thickening. An immediate concern for cardiac amyloidosis was raised. Urine protein electrophoresis and serum protein electrophoresis were consistent with a monoclonal gammopathy. Serum protein electrophoresis showed an M- spike of 2.2 with immunofixation showing IgG lambda monoclonal band. The patient’s troponins lateralized and his lower extremity edema improved with IV furosemide. He was initiated on metoprolol, lisinopril, aspirin, and rosuvastatin and discharged with close hematology and cardiology follow-up.

**Figure 3:**
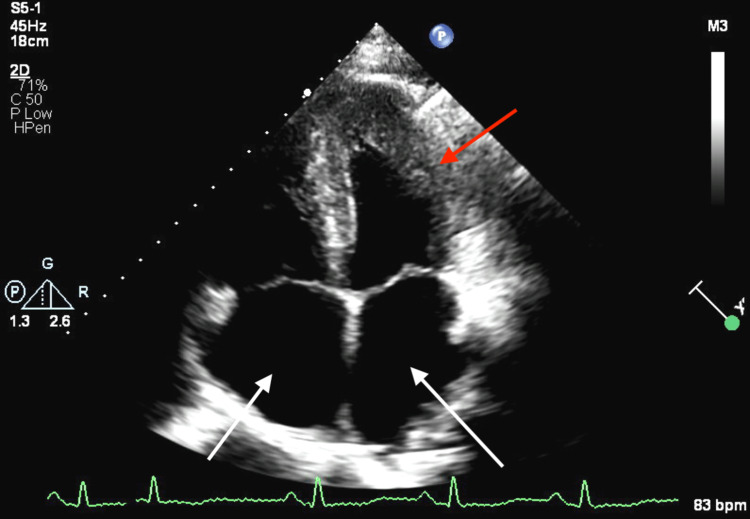
Apical four-chamber view demonstrating left ventricular hypertrophy and biatrial enlargement The red arrow points to left ventricular hypertrophy; the white arrows point to biatrial enlargement.

**Figure 4:**
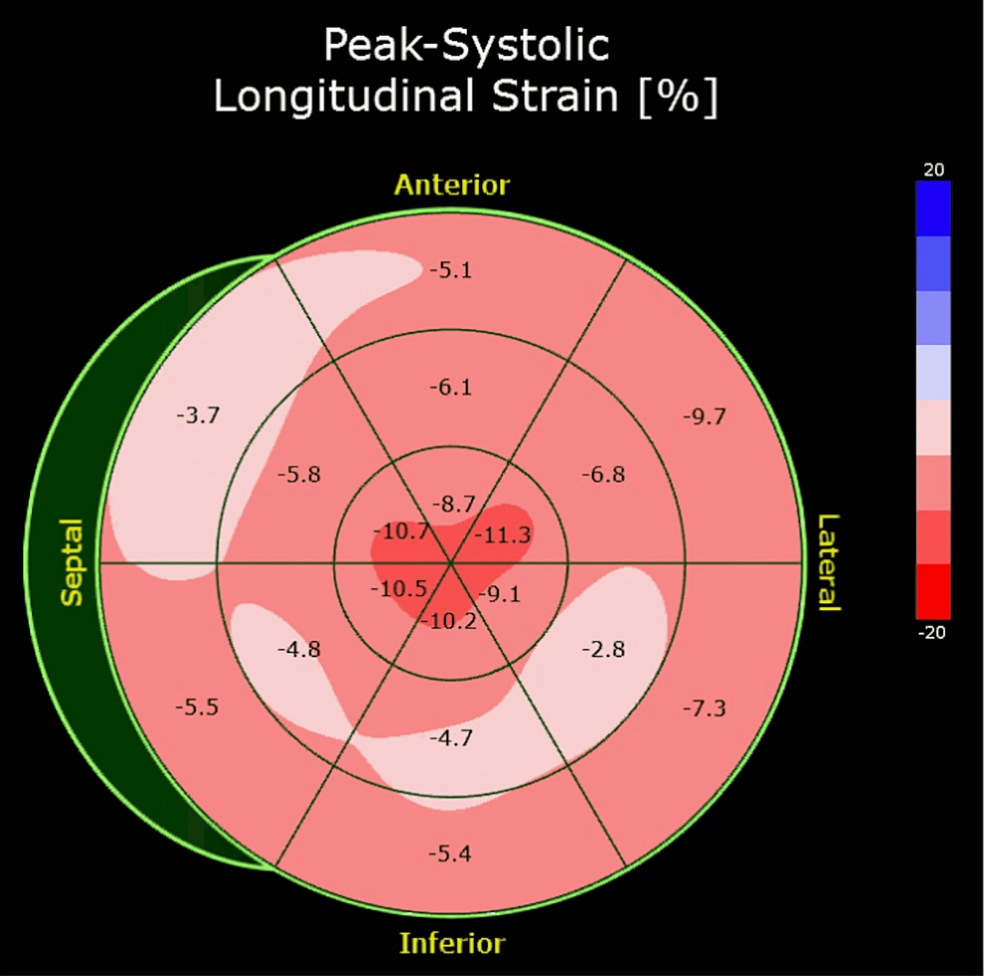
Global longitudinal strain pattern demonstrating apical sparing

The patient was lost to outpatient follow-up but presented to the emergency department with progressively worsening shortness of breath three months after initial discharge. His presentation was consistent with acute congestive heart failure exacerbation in the setting of medication and dietary noncompliance. The patient continued to have elevated troponin I levels similar to prior presentation along with a BNP elevated to 2863 pg/ml and chest x-ray findings consistent with vascular congestion. A repeat TTE demonstrated a reduced LVEF to 35% with severe diffuse hypokinesis, mildly abnormal myocardial specular pattern (Figure 3), and apical sparing of GLS suggestive of amyloidosis (Figure 4). Laboratory workup was also significant for worsening renal function with a creatinine of 1.8 mg/dL but once again negative for calcium or blood count abnormalities. The globulin protein gap remained elevated at 5.1.

Given prior loss to follow-up, the patient underwent an inpatient workup for amyloidosis. His abdominal fat pad biopsy demonstrated fibroadipose tissue with focal Congo red positive vascular deposits consistent with amyloid. A bone marrow biopsy revealed >30% monotypic plasma cells co-expressing CD56 and cytoplasmic lambda light chain. The patient was classified as borderline between smoldering multiple myeloma and plasma cell myeloma by WHO criteria. The patient was initiated on cyclophosphamide, bortezomib, and dexamethasone for the treatment of AL amyloidosis, which can also be used to treat multiple myeloma.

## Discussion

Amyloidosis is a rare systemic disorder characterized by the deposition of insoluble fibrils composed of misfolded proteins in various organs including the kidneys, liver, and heart [3,4]. Amyloidosis is classified based on the nature of the precursor protein that composes the fibrils. Cardiac involvement can be seen in the following types of amyloidosis: AL or primary amyloidosis, transthyretin amyloidosis (ATTR) or familial amyloidosis, systemic senile amyloidosis, isolated atrial amyloidosis, and serum amyloid A (AA) or secondary amyloidosis [5]. AL is the most frequent form with an approximate prevalence of 0.3 cases per 100,000 [6]. Deposition of insoluble amyloid fibrils in the extracellular space of the heart leads to biventricular hypertrophy and decreased ventricular compliance resulting in restrictive cardiomyopathy [7]. Additionally, in patients with AL amyloidosis, amyloid can deposit within or around the cardiac arterioles leading to myocardial ischemia [8]. In addition to restrictive cardiomyopathy, the direct toxic effect of AL amyloid fibrils caused by reactive oxygen species also contributes to the pathophysiology of AL cardiac amyloidosis [9].

It is uncommon for multiple myeloma to present initially as decompensated heart failure. The most common clinical manifestations of multiple myeloma are anemia, infections, bone lesions, and renal failure [10]. Our patient had none of the classical symptoms or laboratory abnormalities commonly associated with multiple myeloma. Given elevated plasma troponin levels and clinical features suggestive of decompensated heart failure, the initial presentation was concerning for acute myocardial infarction (MI). However, an urgent coronary angiogram revealed nonobstructive coronary artery disease not consistent with a type I MI. A high index of clinical suspicion is required to diagnose cardiac amyloidosis. It is our objective to demonstrate the possibility of atypical presentation of multiple myeloma as cardiac dysfunction due to AL amyloidosis. With advancements in echocardiographic technologies and the widespread use of GLS, echocardiography has become a reliable tool for the initial evaluation of cardiac amyloidosis. GLS is derived from tracking myocardial speckles during ventricular systole. A relative apical sparing of longitudinal strain is a sensitive and specific marker of cardiac amyloidosis [11]. A proposed mechanism suggests preferential deposition of amyloid fibrils in the basal and mid segments of the ventricles leading to relative sparing of the apical myocardium [11]. Cardiac MRI imaging can confirm the apical sparing of myocardial contractility, in addition to other features such as biventricular hypertrophy and characteristic distribution of late gadolinium enhancement [11]. In addition to echocardiography, the initial index of suspicion critically depends on electrocardiogram findings. A classic linear relationship between increased left ventricle (LV) thickness and increased ECG voltage is not seen in cardiac amyloidosis [12]. The LV hypertrophy seen on echocardiography in cardiac amyloidosis is driven by amyloid deposition and extracellular matrix expansion as opposed to hypertrophy of electrically active cardiac myocytes. This results in the reduction of EKG voltage as the LV wall thickens in cardiac amyloidosis [12]. Only 25-50% of patients with cardiac amyloidosis meet the low voltage criteria and it should not be regarded as a sensitive marker of the disease [12]. Other EKG findings associated with cardiac amyloidosis include the pseudoinfarct pattern with Q waves, left anterior hemiblock, and rhythm disturbances. It should be noted that none of the EKG findings are sensitive or specific to cardiac amyloidosis [12].

Urine and serum protein electrophoresis remains the gold standard screening test for gammopathies. A definitive diagnostic modality for cardiac amyloidosis remains an endomyocardial biopsy. Even in patients with fat pad biopsy-proven AL amyloidosis, there are reports of co-occurring ATTR amyloidosis in the heart. Unfortunately, access to endomyocardial biopsies remains limited and is still not the standard of care. The importance of correct diagnosis is crucial given the advancements made in the treatment of ATTR amyloidosis with tafamidis.

## Conclusions

Congestive heart failure presentation is ubiquitous in nature. A high degree of clinical suspicion is required to identify relatively rare causes of heart failure. A careful evaluation of electrocardiographic and echocardiographic data is crucial for identifying uncommon causes of heart failure. The diagnostic dilemma is made even more challenging if there are no features to suggest a multisystem disease. Timely diagnosis and initiation of treatment are essential in decreasing mortality in patients with multiple myeloma.
